# INTEGRATION OF DIGITAL TECHNOLOGY IN RESEARCH INVOLVING OLDER ADULTS: LESSONS FROM COMMUNITY-BASED TRIALS

**DOI:** 10.1093/geroni/igac059.825

**Published:** 2022-12-20

**Authors:** Hae-Ra Han

## Abstract

Working with study participants in community-based clinical trials during COVID-19 pandemic has created diverse challenges to study teams. Globally, COVID-related restrictions were implemented including country-wide lockdowns and social distancing, and study teams had to quickly adjust their study protocols to work in a virtual environment. Meeting virtually for recruitment activities or intervention delivery may be particularly challenging when the target group is older adults—one of the vulnerable populations to experience the digital divide due to limited digital access and limited digital literacyThis symposium covers the lessons learned related to use of digital technology in participant recruitment and intervention delivery across a range of populations, including community-dwelling Korean American older adults to African American older women living with pain and low mood, caregivers of persons living with heart failure, and low-income cancer survivors with multiple chronic conditions. The discussion will include 1) findings from screening over 1,000 older Korean Americans to enroll them into a multi-site community-based trial, 2) lessons in diversifying intervention delivery methods to African American older women, 3) the integration of virtual modality into a self-care and social support intervention for caregivers of persons with heart failure, and 4) the deployment of mHealth to deliver a home-based exercise program to ethnically diverse low-income cancer survivors with co-morbid conditions in the setting of the COVID-19 pandemic. This symposium seeks to build the evidence related to recruitment and intervention delivery targeting diverse groups of older adults in community settings using technology by sharing common challenges, experiences, and opportunities.

